# A pilot study investigating the use of 4D flow MRI for the assessment of splanchnic flow in patients suspected of mesenteric ischaemia

**DOI:** 10.1038/s41598-021-85315-1

**Published:** 2021-03-15

**Authors:** Pauline Hall Barrientos, Katrina Knight, Douglas Black, Alexander Vesey, Giles Roditi

**Affiliations:** 1 Clinical Physics, Queen Elizabeth University Hospital, NHS Greater Glasgow & Clyde, Glasgow, G51 4TE UK; 2 Academic Unit of Surgery, Glasgow Royal Infirmary, NHS Greater Glasgow & Clyde, Glasgow, G51 4TE UK; 3 Department of Radiology, NHS Greater Glasgow & Clyde & Institute of Cardiovascular and Medical Sciences, Glasgow, G51 4TE UK; 4 Department Vascular Surgery, University Hospital Hairmyres, East Kilbride, G75 8RG UK

**Keywords:** Diagnosis, Medical imaging, Gastrointestinal diseases, Translational research

## Abstract

The most common cause of chronic mesenteric ischaemia is atherosclerosis which results in limitation of blood flow to the gastrointestinal tract. This pilot study aimed to evaluate 4D flow MRI as a potential tool for the analysis of blood flow changes post-prandial within the mesenteric vessels. The mesenteric vessels of twelve people were scanned; patients and healthy volunteers. A baseline MRI scan was performed after 6 h of fasting followed by a post-meal scan. Two 4D flow datasets were acquired, over the superior mesenteric artery (SMA) and the main portal venous vessels. Standard 2D time-resolved PC-MRI slices were also obtained across the aorta above the coeliac trunk, superior mesenteric vein, splenic vein and portal vein (PV). In the volunteer cohort there was a marked increase in blood flow post-meal within the PV (p = 0.028), not seen in the patient cohort (p = 0.116). Similarly, there were significant flow changes within the SMA of volunteers (p = 0.028) but not for the patient group (p = 0.116). Our pilot data has shown that there is a significant haemodynamic response to meal challenge in the PV and SMA in normal subjects compared to clinically apparent CMI patients. Therefore, the interrogation of mesenteric venous vessels exclusively is a feasible method to measure post-prandial flow changes in CMI patients.

## Introduction

Mesenteric ischaemia comprises a related group of disorders with a variety of aetiologies and natural histories^1–4^. These disorders have not benefitted from anywhere near as much interest and research as other forms of cardiovascular disease, remain relatively poorly understood^5^ and usually have a poor prognosis^6,7^. Diagnosis can be challenging and management strategies variable with patients frequently falling between specialties.

Chronic mesenteric ischaemia (CMI) is an important sub-type of mesenteric ischaemia the incidence of which has been increasing in recent years^8,9^. CMI is almost invariably associated with atherosclerosis. Typically, post-prandial abdominal pain due to induced relative ischaemia (‘mesenteric angina’) and weight loss from food fear combined with a history of atherosclerotic disease in other territories points towards the diagnosis although clinical presentations vary. On the other hand, patients presenting with abdominal pain may be found to have mesenteric atherosclerosis on computed tomography (CT) prompting uncertainty over whether the atherosclerosis is the causative pathology for the presentation or just a bystander phenomenon; this is because the mesenteric circulation is highly collateralised such that arterial occlusions are commonly asymptomatic^10–13^. As such, there is often diagnostic uncertainty when faced with a patient in whom CMI is part of the differential diagnosis, in some cases of diagnostic doubt then invasive treatments such as angioplasty and stent may be offered on a speculative basis, not ideal given the potential for the significant morbidity and even mortality associated with such interventions. Therefore, techniques that are capable of reliably demonstrating both anatomic arterial steno-occlusive disease and any consequent down-stream impairment of perfusion and ideally inducible reversible ischaemia (analogous to the use of myocardial perfusion techniques for the coronary circulation) will provide the best path to appropriate diagnosis and hence decisions about treatment. However, such techniques are not yet in routine clinical use.

### Assessing anatomy and inducible relative ischaemia

Duplex ultrasound of the mesenteric arteries is a reasonable first line test in patients where there is a low but definite suspicion of CMI. In thin subjects, Duplex can be an excellent test to evaluate arterial stenosis where the vessel is well visualised but there is much interobserver variability as for many ultrasound vascular evaluations and often imaging is limited anatomically to the superior mesenteric artery (SMA) alone. While an increase in velocities across a stenosis after meal challenge point to the diagnosis of true mesenteric angina the actual bulk flow rates are not evaluated and collateral circulations are not assessed. Furthermore, to assess for haemodynamic change the imaging is very often compromised by bowel gas and peristaltic motion following meal challenge.

Computed tomography angiography (CTA) offers high-resolution 3-dimensional arteriography and an assessment of the solid and hollow abdominal viscera^14–17^. Vessel suitability for endovascular intervention or operability may be assessed, as may any collateral circulation. However, the imaging is static and only offers a very rough assessment of likely perfusion and potential for induced relative ischaemia. In the current absence of alternative single-phase CTA is thought a reasonable test for most patients with suspected CMI.

Magnetic resonance imaging (MRI) and magnetic resonance angiography (MRA) also provide non-invasive alternatives to conventional arteriography and CTA for patients with suspected CMI. Contrast-enhanced magnetic resonance angiography (CE-MRA) can clearly map arterial steno-occlusive disease and collateral pathways similar to CTA, the lack of ionising radiation allows for multiphase and dynamic examinations without radiation penalty allowing evaluation of arterial and venous structures and even providing qualitative flow information. A potential further benefit of a MRI approach is that it is capable of delineating not only the vascular anatomy but with the addition of flow sensitive techniques also the flow dynamics in a single test^18^. MRI is now readily available in vascular centres and has become the imaging modality of choice in the peripheral circulation supported by evidence-based guidelines^19,20^. There have been previous studies in the 1990s^21–23^ where mesenteric flow response to meal challenge (in the superior mesenteric arteries and veins) was studied using 2D phase contrast imaging (2D time-resolved PC-MRI), however, these studies had a limited number of patients and volunteers. A significant limitation of the use of 2D PC-MRI that has impeded its clinical uptake is the need for accurate planning of acquisition planes orthogonal to what can be small, tortuous and variably angled vessels. 4-dimensional flow sequences have been developed, enabling haemodynamic measurement within a single volume of interest. This potentially greatly simplifies the acquisition of flow data since a single relatively large volume encompassing all vessels of interest can be simply placed without specific regard to individual vessel orientations etc. However, its use in the investigation of GI responses to situations of demand, such as the post-prandial phase, in both healthy patients and those with symptoms suggestive of chronic mesenteric ischaemia remains under-investigated.

Interrogating the portal vein (PV) and/or the superior mesenteric vein (SMV) is likely to be the optimal method of quantifying mesenteric blood flow as in most situations, the entire circulation of the gut will pass through this single large vessel. On the other hand, measuring and summing flow through multiple small, diseased, tortuous and collateralised arteries is challenging even with a single acquired volume and unlikely to be as reproducible. In addition, the PV is virtually never calcified and is rarely stented; both situations which can lead to signal loss in MRI. In very heavily calcified arteriosclerotic disease there may be such dense calcification that arterioliths are formed and the calcium density can create a local susceptibility artifact.

The aim of this prospective pilot study was to determine the feasibility of assessing the response of the mesenteric vasculature to a meal stimulus using 3D time-resolved PC-MRI, also known as 4D flow, in both healthy participants and patients with symptoms characteristic of CMI. This study was also designed to compare haemodynamic assessment of the venous versus arterial mesenteric systems. 4D flow data were also compared to established 2D time-resolved PC-MRI, known as 2D flow, techniques for validation.

## Materials and methods

### Subjects

A total of twelve participants were recruited, 6 patients and 6 healthy volunteers. The patients (mean age 65 y, range 44–81 y, 2F), with characteristic CMI syndromes were identified with clinical consensus from vascular multidisciplinary team meetings (MDTM) comprising consultant vascular surgeons and interventional radiologists, at the Queen Elizabeth University Hospital, Glasgow and University Hospital Hairmyres, East Kilbride. Patients were recruited with clinically obvious CMI based on a combination of history and physical examination plus all their imaging features from standard investigations. Patients where there was any clinical doubt as to the aetiology of their abdominal pain were not included in the study.

Healthy subjects (mean age 39 y, range 30–77 y, 4F) were recruited from the Glasgow Clinical Research Imaging Facility (CRIF) volunteer bank. Inclusion criteria were volunteers over the age of 18 years with no known mesenteric ischaemia symptoms or other gastrointestinal disorders, no clinical history of vascular disease and no clinical signs of peripheral vascular disease. Subjects with arrhythmia or MR non-conditional implants and devices were excluded.

### Statement

This study was approved by the local ethics committee, Glasgow CRIF approval group, and written informed consent was obtained from all participants. Research Ethics Committee West of Scotland REC3, reference 16/WS/0220. All experiments and methods were performed in accordance to relevant guidelines and regulations. We applied the following methods, meal challenge and MR imaging protocol and data analysis, from Roldán-Alzate^24^ to measure flow changes in arterial and venous vessels post-prandial.

### Meal challenge

For the baseline scan all participants were asked to fast for 6 h (no food, liquid or chewing gum). After the initial scan subjects were asked to consume a standard meal, 400 mL of Ensure Plus (700 cal) and were then scanned again 60 min post meal ingestion. 60 min was selected as this was quoted to be the peak time of blood flow within the SMA post meal returns to baseline after 2–3 h, which coincides with CMI patients experiencing pain lasting 1–2 h after eating^25,26^. In addition it was felt that is was appropriate to scan post-prandial at 60 min, unlike Roldán-Alzate, due to the fact that within 20 min post-prandial one cannot guarantee that a significant flow response could be measured since the rate of change of blood flow can vary from person to person^27^. All patients and volunteers, apart from two, were scanned between 9 to 10 a.m. to account for diurnal changes. Due to logistics one patient and one volunteer were scanned at 2 p.m.

### MR imaging

All scans were performed using a 3.0T Prisma MRI system (Siemens Healthineers) with an 18 channel body array coil. Both 4D flow and 2D flow data were acquired for each of the subjects.

Arterial and venous vessels were imaged using: True FISP fat saturated breath held, TR/TE 246/1.31 ms, FoV 340 mm, resolution 1.3 × 1.3 mm, slice thickness 2.4 mm, flip angle 36°, TA 20 s in the transverse, sagittal and trasnsverse planes. Additional images were acquired of the PV and SV vessels to facilitate perpendicular planning of 2D flow slices across these vessels, since they were not easy to plan from the True FISP fat saturated images. The following sequences were used True FISP single shot fat saturated breath held, orthogonal to the vessel, FoV 400 mm, TR/TE 986/3.26 ms, acquired resolution 1.6 × 1.6 mm, slice thickness 4 mm, flip angle TA 20 s. Followed by a breath held True FISP cine planned perpendicular to the middle of the imaged vessel, FoV 340 mm, resolution 1.3 × 1.3 mm, slice thickness 7 mm, flip angle 44°, TA 4.7 s. The cine sequence would confirm the pulsatile motion of the PV or SV vessel and was then used for the positioning of the 2D flow scans.

2D flow data were acquired, pre- and post-prandial, across the aorta at levels above the coeliac artery, above (SMA was also visualised in this plane) and below the renal arteries, planned using HASTE images. The superior mesenteric vein (SMV), splenic vein (SV) and portal vein (PV) were planned as described from the section above. The 2D time resolved PC-MRI data were acquired with the following parameters: resolution 1.8 × 1.8, slice thickness 6 mm, TR/TE 3.7–5.3/2.47–3.1 ms, with a scan time of 14 s (breath-hold) and 30 time frames between each R-R interval. The velocity encoding value (Venc) was set at 50 cm/s for the veins and 150 cm/s for the arterial planes with option to increase Venc if aliasing was apparent. The acquisitions used retrospective ECG gating.

The research 4D flow sequence (WIP 785A, the product is currently under development and is not for sale in the US and in other countries. Its future availability cannot be ensured), from Siemens, acquired the data with the following imaging parameters: imaging volume 288 × 288 × 72 mm, 1.8 mm acquired resolution, 1.8 mm slice thickness, TR/TE = 4.8–5.9/ 2.25–3.19 ms, iPat 3, with a scan time of ~ 8 min and 20 time frames between each R-R interval. The acquisition used retrospective ECG gating and navigators (placed over liver/diaphragm) for respiratory gating, with an acceptance window of ± 8 mm. Two volumes of 4D flow data were acquired. First 4D flow acquisition was for the SMA with the Venc set at 150 cm/s (pre- and post-meal) for volunteers and 200 cm/s (pre-meal) and 220 cm/s (post-meal) for patients. A higher Venc setting was selected for patients to account for potential jet flow distal to any SMA stenosis.

The second 4D flow acquisition was for the PV. 2D flow was acquired initially to help determine the Venc settings for 4D flow of the PV. Therefore, for 4D flow the Venc was set at 30 cm/s (pre-meal) and 40 cm/s (post-meal) for volunteers and 20 cm/s (pre-meal) and 30 cm/s (post-meal) for patients. A lower Venc setting was selected for patients as it was found from 2D acquisitions that these patients typically had a lower peak velocity when compared to the healthy volunteers. It was crucial that the Venc set for both arterial and venous flow matched the real velocity within the vessel, if this was not done then the region of interest was acquired with a lower, less optimal signal to noise ratio (SNR)^28^. However, it can be challenge to find a Venc setting that will image both slow and high velocities through and distal to the SMA stenosis, therefore a higher Venc was selected to measure the jet flow distal to the stenosis. Venc of 220 cm/s was able to visualise this sufficiently.

The order of the flow sequences was as follows for all patients and volunteers post-meal:

- Localisers and anatomical images
- PV 2D cine PC MRI
- PV 3D cine PC MRI
- SMA 3D cine PC MRI (during this SV, SMV, arterial flow measurements were planned)
- Remaining arterial and venous 2D cine PC MRI

Post-flow PV 2D and 3D cine PC were acquired ~ 60 min after the patient had finished their meal. Therefore SMA flow measurements were acquired ~ 70 min post meal.

### 2D and 4D flow MRI data analysis

2D time-resolved PC-MRI data were analysed using Argus Flow software (Siemens Healthcare GmbH, Erlangen, Germany). Anatomical, magnitude and phase images were imported into Argus Flow and background phase and phase anti-aliasing (10% of highest Venc) corrections were applied. A region of interest was selected over the visible lumen. The software then calculated the following waveforms and indices: area, flow, mean velocity and peak velocity.

4D flow images were processed using Siemens prototype software (Flow version 2.4, Flow version 2.4, Siemens Healthcare GmbH, Erlangen, Germany). Data sets were uploaded to the software and corrections were applied onto the data set. These included: phase anti-aliasing, background phase correction, and motion tracking.

**Figure 1:**
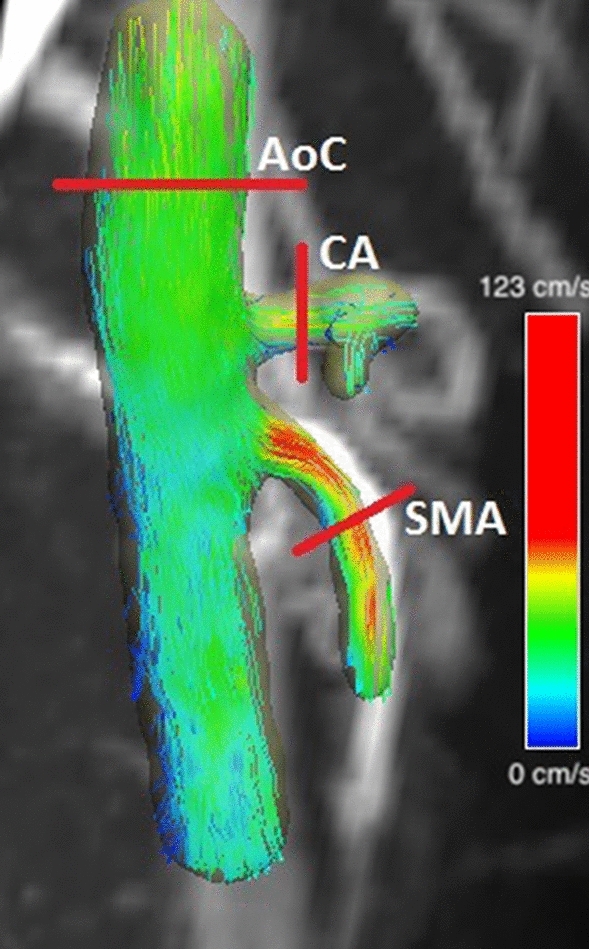
Segmentation of arterial vessels with planes of analysis annotated.

**Figure 2:**
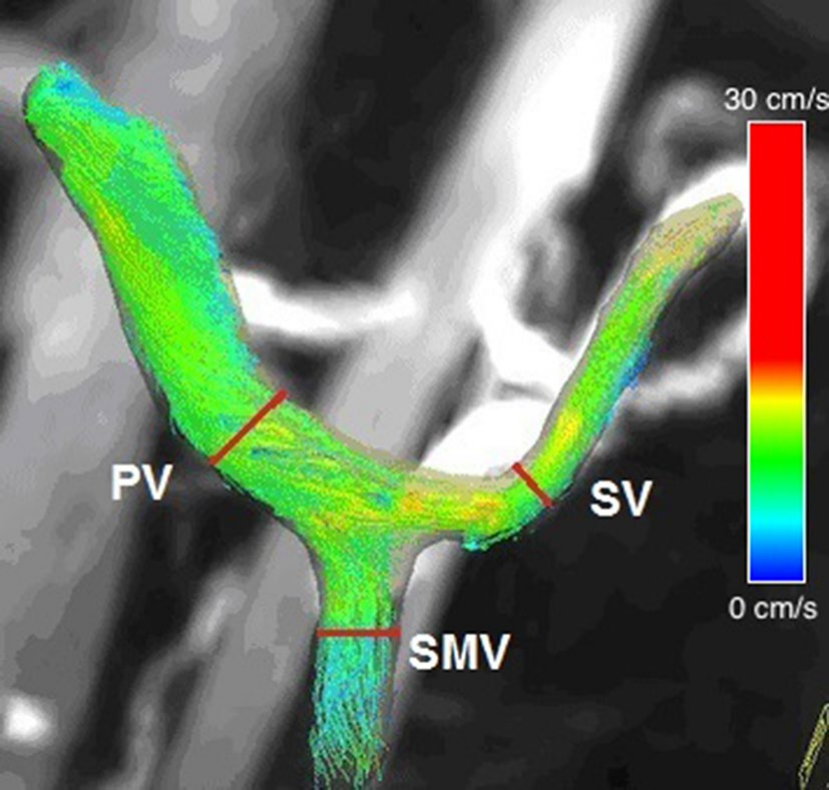
Segmentation of venous vessels with planes of analysis annotated.

Vessels were segmented using a centreline model. The vessels identified from the two data sets, for both pre- and post-meal, were the visceral aortic segment and the portal venous system, as shown in Figs. 1 and 2. Analysis planes were selected manually and placed in the following regions for each patient and volunteer:

1. Aorta above coeliac trunk (AoC)
2. SMA (for patients then beyond any stenosis)
3. Coeliac artery (CA)
4. PV (~ 1 cm distal to the confluence)
5. SV
6. SMV

For each of the planes selected quantitative parameters were automatically generated by the Siemens software. These parameters were a time integrated flow evaluation which included maximum peak velocity magnitude, temporal average net flow, temporal average net forward volume within the contour over time. The software also generated 3D streamlines within the segmented vessels as shown in Fig. 1. The total time for the data to be processed was ~ 15 min. 4D flow slices were matched to the 2D slices. This was done by identifying the x, y and z patient coordinates of the centre of 2D flow vessel using Osirix Lite. Several analysis planes were then prescribed across the vessels in the 4D flow software. The coordinates of these 4D planes were then matched to the 2D. Unfortunately, the coordinates of the 4D slices are only known after the results are exported, if the coordinates did not match further analysis planes were added in the 4D flow software. The inter-operator variability was tested using Bland–Altman analysis for both 2D and 4D. A total of 2 appraisers and 36 vessels from 6 data sets were used. The average flow through the vessel of the PV, SMV, SV, AoC, SMA were measured.

For internal consistency of flow measurements, the conservation of mass was tested for each subject at the portal confluence, this was determined for both pre- and post-prandial separately. This was done by measuring the flow in the PV, SV and SMV such that:

1 $$QPV = QSV + QSMV$$

where QPV, QSV and QSMV is flow in the PV, SV and SMV respectively.

### Statistics

**Figure 3:**
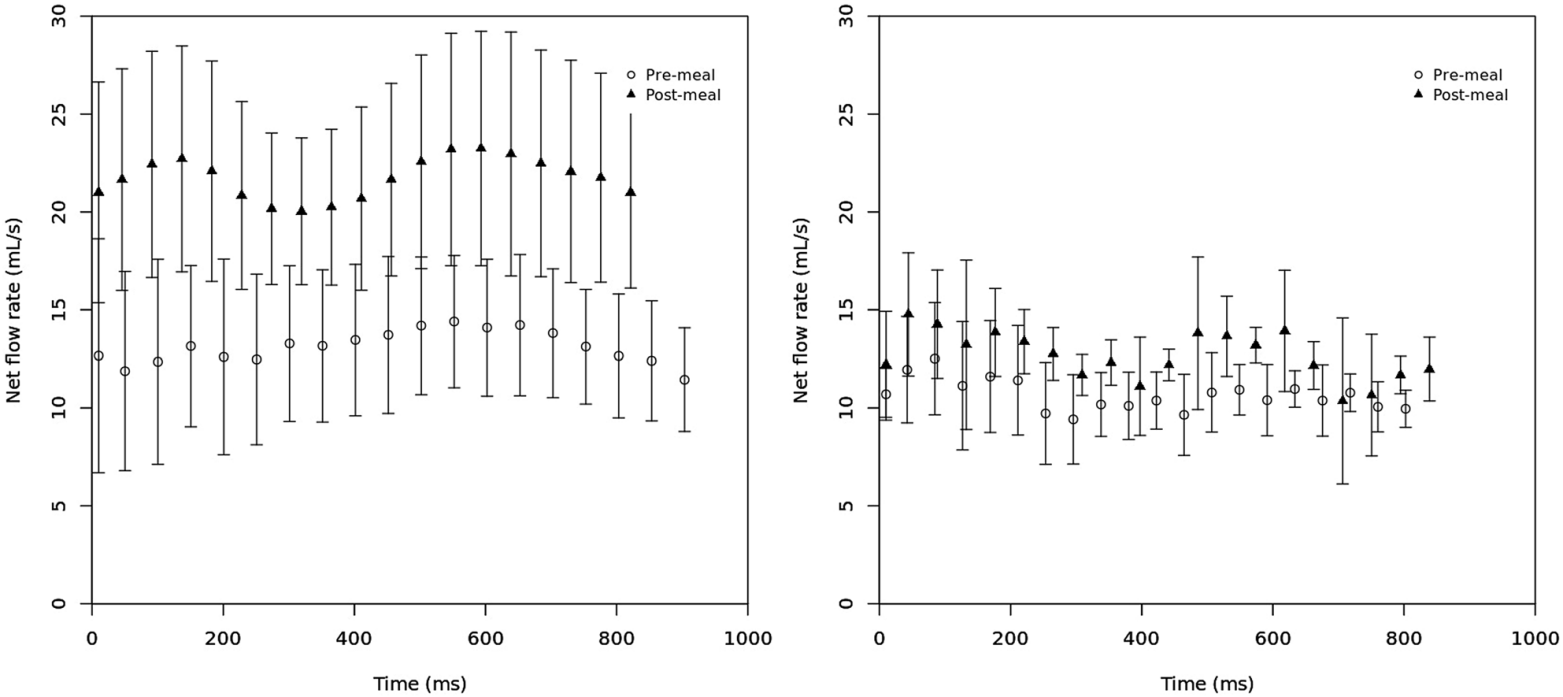
Average net flow through the PV for volunteers (left) and patients (right). Error bars indicate the standard deviation. Plot created using R.

**Figure 4:**
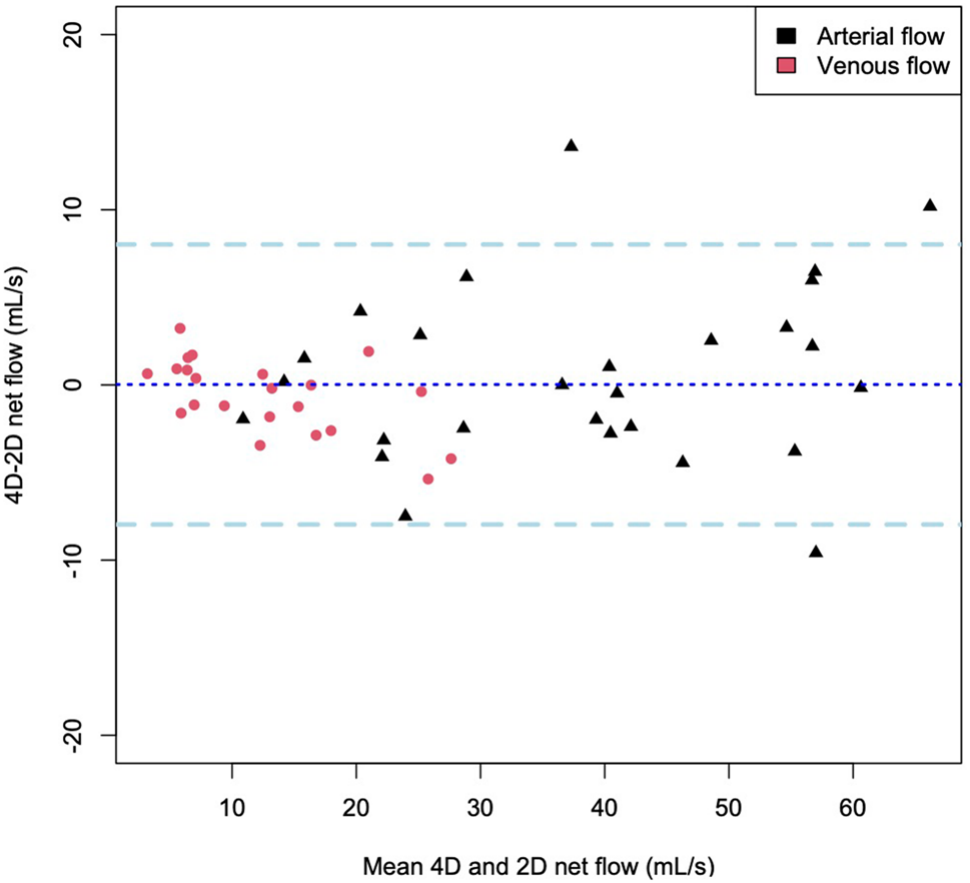
Bland–Altman results of 2D and 4D average flow for all analysis planes. Plot created using R.

Flow values between 2 and 4D flow and changes between pre- and post-meal were studied using Wilcoxon signed rank test (2-tailed). The statistics package IBM SPSS Statistics for Windows, version 24 (IBM Corp., Armonk, N.Y., USA). Figures 3 and 4 were created using R (R Core team 2017), using package ggplot 2.

## Results

### Volunteers

In the 6 volunteers, the mean fasted average net flow rate in the portal vein was 13.4 ± 3.9 mL/s. There was a significant increase in flow observed after the standardised meal increasing to 19.5 ± 4.7 mL/s p = 0.028 (Fig. 3). This was predominantly due to flow in the SMV which was increased (p = 0.046) whereas flow in the SV and CA was not significantly different (p = 0.917, p = 0.173 respectively).

The mean fasted average net flow rate in the SMA was 5.8 ± 1.5 mL/s. There was a significant increase in flow observed after the standardised meal to 11.0 ± 2.9 mL/s (p = 0.028). In the AoC there was also a significant increase (p = 0.043). Table 1 details the full results for the volunteers.

**Table 1:** Average flow results in volunteer cohort.

Average net flow (mL/s)	PV	SMV	SV	AoC	SMA	CA
Pre-meal	13.4 ± 3.9	6.1 ± 1.5	7.1 ± 1.5	49.1 ± 8.5	5.8 ± 1.5	12.2 ± 3.3
Post-meal	19.5 ± 4.7	11.3 ± 3.2	7.6 ± 1.2	60.2 ± 13.0	11.0 ± 2.9	10.0 ± 2.9
P value	0.028	0.046	0.917	0.043	0.028	0.173

*PV* portal vein, *SV* splenic vein*, SMV* superior mesenteric vein, *AoC* aorta at coeliac artery, *SMA* superior mesenteric artery, *CA* coeliac artery.

### Patients

For the 6 patients the mean fasting average net flow rate in the portal vein was 10.2 ± 2.1 mL/s. After the standardised meal there was not a statistically significant increase in flow in the portal vein: 11.5 ± 1.6 mL/s (p = 0.116) (Fig. 3). Flow in the SMV and SV did not significantly change, p = 0.075 and p = 0.5 respectively.

The mean fasted average net flow rate in the SMA was 5.3 ± 1.5 mL/s. An increase in flow after the standardised meal was apparent 7.8 ± 2.2 mL/s (p = 0.116), but this was not statistically significant for this small number of patients. Similarly, there was an apparent increase in flow at the supra-coeliac aorta but this also did not reach statistical significance (p = 0.173). Table 2 details the full results for the patient cohort. In one case the SV could not be identified due to insufficient velocity sensitivity. In only four patients was the CA clearly identified and in the other two this vessel was stenosed. These results were not included in the final analysis.

**Table 2:** Average flow results in patient cohort.

Average net flow (mL/s)	PV	SMV	SV (n = 5)	AoC	SMA
Pre-meal	10.2 ± 2.1	5.7 ± 1.7	5.7 ± 1.5	44.9 ± 12.1	5.3 ± 1.5
Post-meal	11.5 ± 1.6	6.2 ± 1.8	5.4 ± 1.4	48.4 ± 10.9	7.8 ± 2.2
P value	0.116	0.075	0.5	0.173	0.116

*PV* portal vein, *SV* splenic vein*, SMV* superior mesenteric vein, *AoC* aorta at coeliac artery, *SMA* superior mesenteric artery.

### 2D vs. 4D flow

Figure 4 demonstrates the Bland–Altman results for 4D flow compared to 2D flow for all analysis planes. There is a good agreement between 4 and 2D net flow measurements with a mean bias of 0.02 ± 8.16 mL/s (p = 0.633).

### Interobserver variability

For both 2D and 4D methods the overall the inter-observer variability was good, mean bias 0.07 ± 3.40 mL/s (p = 0.816) and mean bias − 0.72 ± 6.27 mL/s (p = 0.195) respectively.

### Quality control

For all subjects we found that the conservation of flow within the portal vein was met within reasonable limits. The average flow difference between QSV-QSMV and QPV for volunteers was 0.2 ± 1.7 mL/s (percentage difference 0.6 ± 13%) and 0.5 mL/s (percentage difference 1.6 ± 7.9%) for pre- and post-prandial respectively. For patients the average flow difference was 2.1 ± 4 mL/s percentage difference 3 ± 13%) and 2.2 ± 3.4 mL/s (percentage difference 8 ± 8%) for pre- and post-prandial respectively.

## Discussion

This pilot study measured the haemodynamic response in both the venous and arterial vasculature of 6 patients with clinically apparent CMI and 6 healthy volunteers. Baseline and post-prandial flow changes in both the PV and SMA have been quantified. Our data demonstrate that there is a significant measurable hemodynamic response to a standard meal challenge within the PV, SMV and SMA in healthy volunteers whereas in the patient cohort this normal physiological response was completely attenuated.

Although there are no previous studies using 4D flow on CMI patients there has been research in PV flow changes, post-prandial, in the setting of patients with portal hypertension and healthy volunteers by Roldán-Alzate et al.^26^. This group showed significant flow changes in the PV in healthy volunteers and minimal flow changes in their patients with portal hypertension. However, their measurements of post-prandial flow were taken 20 min post ingestion. We have demonstrated that these blood flow changes can be detected 60 min after eating a meal. However, this does not justify that for a larger study one should use 60 min to study blood flow changes post meal. Unfortunately, there is conflicting guidance of timings to detect changes and a lack of research in this area^27,29^. Therefore, we recognise that we need to have a larger in-depth study of blood flow changes post-prandial over a period of time. For example, measure blood flow over a period of 90 min, every 15 min, for different age groups.

Our pilot study, for both volunteers and patients, were not aged matched limiting our knowledge of ‘normal’ flow responses across different age groups. Nor we have not accounted for severity of disease progression as we had identified patients as having a stenosis present. Therefore, it is pertinent to determine the optimal time for mesenteric blood flow changes in not only older patients but to account for varying degrees of vessel disease, number of stenoses, presence of collateral pathways and symptomatic pain. This pilot study did not investigate wall shear stress of both arterial and venous vessels for both patients and volunteers, since the sequences were optimised for blood flow. A study of wall shear stress could also be investigated, as it is well known that elasticity will change with age which could also influence blood flow responses^30^.

Another limitation of our study was the direct comparison of flow scans and its timings. We ensured that our PV flow were prioritised, 2D and 3D cine PC, and measurements were acquired 60 min post-prandial. However, because of this it pushed the arterial measurements beyond this time point. Because of this we may not capture a significant blood flow change in these vessels for both patients and volunteers. However, flow changes in the SMA flow changes were measured ~ 70 min in volunteers, which indicates one can could still detect flow changes > 60 min. To confirm this a blood flow study, as mentioned, would be needed.

Similar to Roldán-Alzate’s study we also found the portal vein interrogation to be reliable and reproducible and conservation of flow into the portal vein from the major tributaries was demonstrated. We also did not observe any temporal order changes in 4D flow acquisition post-prandial. Therefore, in future studies, we contend that only PV 4D flow data need be acquired. This will reduce scanning time and improve acceptability for the patient. A separate MR arteriogram can be obtained with or without contrast before or after the flow study for anatomy and intervention planning. As discussed in the introduction, portal vein flow is an acceptable parameter of overall mesenteric flow being a single, large easily identified vessel with the additional advantage of being (almost always) free from stents, calcification and stenosis meaning the resulting data will not be subject to artefacts due to metalwork or jet velocities.

The pilot study also validated the results of 4D flow using 2D flow showing agreement between 4 and 2D measurements. However, there are several outliers, in particular with a net flow > 40 mL/S. This was because 4D flow planes did not match the selected 2D slices, measurements above the coeliac artery and below the renal vessels were the main issues. This was because a few of the 2D slices were acquired out with the 4D volume. This highlights the main limitation of 2D flow-data analysis is limited to one position and it cannot changed post-hoc. 2D flow acquisition is time-consuming with slice planning being challenging and prone to inter-operator variability. We found on average (including planning and repeat scanning if required) to acquire 6 2D flow measurements took an average of 20 min. We found that scans are often repeated, in both planning and flow imaging, due to poor breath holding by the patient/volunteer or due to the challenging nature of planning small vessels. In particular, MRI technical staff found it difficult to identify and carefully plan slices across the vessels for patients with little visceral fat, this is readily overcome by volumetric 4D flow acquisitions. However, 2D flow was useful in this pilot study to determine the optimal Venc setting for 4D flow, if Venc setting was > 10% than the peak velocity the acquired 4D flow data was subjected to a lower SNR (particularly noticeable in subjects with increased subcutaneous and/or visceral fat.). However, from this study we can determine the optimal Venc for future PV studies, 30 cm/s or pre- and 40 cm/s for post-prandial, for both patients and volunteers. Therefore, for larger studies one would no longer require 2D flow and its additional anatomical images, giving the freedom to interrogate the whole PV rather than one 2D slice, which cannot be fully guaranteed to be reproducible in other patients. Currently the main disadvantage is the acquisition time for 4D flow acquisitions are quite long, on average 8 min. Hopefully accelerated sequences such as kt-acceleration, non-Cartesian under sampling (radial, spiral) and compressed sensing will overcome this limitation^31,32^. A further limitation is that if arterial data is desired then a second 4D flow acquisition is currently required as we did in this pilot study, in the future dual-Venc 4D flow sequences may obviate this limitation also although we contend that subject to further validation PV flow alone should suffice for diagnosis or refutation of suspected CMI^33^.

## Conclusion

We acknowledge that this pilot study was necessarily small and that a larger aged matched controlled cohort study and taking into account the presence of diseased vessels will be required to further validate our findings. In addition, a scan-scan repeatability study would need to be undertaken to determine the precision and reliability of 4D flow for the mesenteric vasculature. This study will provide the justification for this technology assessment.

This work has shown MRI 4D flow holds promise in patients suspected of CMI and is a feasible alternative to the combination of CTA and Duplex ultrasound in providing both anatomic and functional data from a single scan without the limitations inherent in the current conventional techniques.
